# Left Ventricular Remodeling and Heart Failure Predictors in Acute Myocardial Infarction Patients with Preserved Left Ventricular Ejection Fraction after Successful Percutaneous Intervention in Western Romania

**DOI:** 10.3390/life12101636

**Published:** 2022-10-19

**Authors:** Diana-Aurora Arnautu, Minodora Andor, Bogdan-Flaviu Buz, Mirela-Cleopatra Tomescu, Cristina Vacarescu, Simina Crisan, Dan Gaita, Constantin-Tudor Luca, Dragos Cozma

**Affiliations:** 1 Department of Cardiology, Faculty of Medicine, “Victor Babes” University of Medicine and Pharmacy, 2 Eftimie Murgu Sq., 300041 Timisoara, Romania; 2 Institute of Cardiovascular Diseases Timisoara, 13A Gheorghe Adam Street, 300310 Timisoara, Romania; 3 Multidisciplinary Heart Research Center of the “Victor Babes” University of Medicine and Pharmacy, 12 Revolution of 1989 Bd., 300040 Timisoara, Romania; 4 Cardiology Clinic of the Timisoara Municipal Clinical Emergency Hospital, 12 Revolution of 1989 Bd., 300040 Timisoara, Romania

**Keywords:** AMI, PCI, infarct-related longitudinal strain, LV remodeling, heart failure hospitalizations

## Abstract

**Simple Summary:**

Acute myocardial infarction patients are at high risk for heart failure, even after successful revascularization therapy. We performed a study that aimed to identify early predictors of heart failure after myocardial infarction. The study included 244 patients discharged with a normal heart function after their first myocardial infarction. At the 2- year follow-up, we found that 186 (76%) showed an improvement in left ventricular function and 61 (24%) had a deterioration of the latter. Comparing the two groups, we found patients in the latter group were older, more often hypertensive, smokers, and had more severe clinical presentations of the acute myocardial infarction and more extensive coronary disease. A total of 19 patients (8%) had experienced hospitalizations for heart failure, 6% being from group I and 12 % from group II, and this difference was notable. We observed that the presence of heart failure at the admission for acute myocardial infarction and abnormal deformation patterns of the infarct-related myocardial segments detected by echocardiography accurately predicted the occurrence of heart failure during the next two years. The presence of these parameters might enable a better risk-stratification and initiation of an effective preventive therapy in acute myocardial infarction patients.

**Abstract:**

(1) Acute myocardial infarction (AMI) patients are at risk of left ventricular (LV) remodeling and heart failure (HF), even after successful revascularization by percutaneous coronary intervention (PCI). We wanted to assess the independent predictors of these outcomes in AMI patients. (2) Methods: The study enrolled patients with a LVEF ≥50% after a successful PCI for their first AMI. After 24 months, patients were separated into two groups based on whether their LVEF remained ≥50% (group I), or decreased to <50% (group II). (3) Outcomes: 26% of the patients experienced a decrease in LVEF below 50%, 41% showed LV remodeling, and 8% had experienced HF hospitalizations. HF hospitalizations were significantly more frequent in group II patients (*p* < 0.0001). The Killip class at admission >2, infarct-related longitudinal strain ≤−12.5%, and the presence of LV remodeling were identified as independent predictors of HF hospitalizations. (4) Conclusions: About 26% of AMI patients with normal LV function after a successful PCI developed HF. More sensitive techniques are required that allow for a more efficient risk-stratification and preventive therapy to reduce LV remodeling and HF in AMI patients with LVEF ≥50% after a successful PCI. The detection of abnormal ventricular deformation patterns after PCI by speckle-tracking echocardiography might be a valuable method in this approach.

## 1. Introduction

Left ventricular (LV) remodeling after acute myocardial infarction (AMI) treated with primary percutaneous coronary intervention (PCI) is a major determinant for both short-term and long-term clinical events [1]. It implies alterations in ventricular architecture affecting the infarcted as well as the non-infarcted segments, followed by an ongoing increase in LV systolic and diastolic volumes [2], and it has been linked to heart failure, arrythmias, and increased mortality.

Although primary PCI has drastically improved the outcomes of AMI patients, detrimental left ventricular (LV) remodeling still occurs in 30% of them [3].

It is well recognized that traditional echocardiography is the preferred imaging approach for the detection of heart failure (HF) in AMI patients, by evaluating the left ventricular (LV) ejection fraction (EF) and the score index of wall motion (WMSI) [4]. Left ventricular strain (S) and its strain rate (SR) evaluated by two-dimensional speckle tracking echocardiography (2D-STE) are newer and more effective methods of estimating myocardial performance, capable of highlighting subtle changes in LV function, particularly in patients with maintained LVEF [5]. In AMI patients, speckle-tracking echocardiography with the evaluation of global longitudinal strain (GLS) and of infarct-related longitudinal strain (ILS) represent a more sensitive approach than LVEF for detecting subclinical LV dysfunction and to estimate the infarct size [6].

It is essential to evaluate all AMI patients for risk in order to properly target them for treatments that may improve the outcome. The aim of this research was to identify independent predictors of LV remodeling and heart failure in adults with acute myocardial infarction and preserved LVEF following successful myocardial revascularization via PCI.

## 2. Materials and Methods

### 2.1. Patient Selection

This is a case-control observational study that enrolled all sequential patients hospitalized with a first acute myocardial infarction (STEMI/high-risk NSTEMI) between January 2019 and August 2021, who were in sinus rhythm and had a LVEF ≥50% after a successful PCI performed in the first 12 h after the onset of symptoms. Patients were high-risk NSTEMI and met ≥1 of the subsequent criteria: a Global Registry of Acute Coronary Events (GRACE) score >140, dynamic alterations in the ST-segment/T wave, or a relative increase or decline in cardiac enzymes [7]. Patients who presented with high-risk NSTEMI and could not undergo PCI during the first 12 h of symptom onset were removed from the study.

The research was carried out at the Cardiology Clinics of the Timisoara Institute of Cardiovascular Disease and of the Emergency Municipal Clinical Hospital of Timisoara. Patients with old myocardial infarctions and those with severe valvular disease were excluded. Conventional and 2D-spackle tracking echocardiography were performed at baseline (immediately following PCI) and after 24 months. Infarct-related segments were identified as those with longitudinal strains (LS) below −15% [8].

According to the LVEF measured at 24 months after the AMI, the patients were divided into two groups based on whether the LVEF was ≥50% (group I), or <50% (group II).

The follow-up period was 24 months, and the endpoints were cardiovascular deaths and hospitalizations for heart failure.

The study was authorized by the Ethics Committee of the Victor Babes University of Medicine and Pharmacy in Timisoara. In compliance with the Helsinki Declaration, all patients signed an informed consent for involvement in the research.

### 2.2. Data Extraction

Age, gender, risk factors for cardiovascular disease (diabetes mellitus, obesity, hypertension, smoking status, hypercholesterolemia), physical examination data, laboratory results, resting electrocardiogram in 12 leads, transthoracic echocardiography, and coronary angiography were acquired from the initial hospitalization for the AMI. During the follow-up period of 24 months deaths, hospitalizations for heart failure, repeated PCIs, and CABGs were recorded.

### 2.3. Definition of Covariates

STEMI was diagnosed when a minimum of two of the following criteria were present: (1) typical angina lasting more than 20 min; (2) ST-segment elevation of ≥1 mV lasting over 0.08 s starting from point J in at least two neighboring leads; and (3) transient rise in cardiac enzymes to more than twice the common laboratory level [9]. NSTEMI was defined when typical angina lasted more than 20 min at rest, there was a temporary increase in cardiac enzymes to more than double the common laboratory levels value, and no ST- segment elevation was noted [10].

According to their clinical presentation, the AMI patients were allocated to one of the Killip classes: Class 1—no heart failure; Class 2—mild heart failure (high jugular venous pressure, a third heart sound present, limited rales in the lungs); Class 3—severe heart failure, acute pulmonary edema (elevated jugular pressure, a third heart sound present, restricted rales in the lungs); Class 4—cardiogenic shock (SBP less than 90 mmHg, signs of peripheral vasoconstriction, including cyanosis, oliguria, or sweating) [11].

### 2.4. Serum Biomarkers

Peripheral plasma samples were taken at the time of admission, within 24 h before echocardiography. NT-proBNP was measured immediately after blood sampling using a Modular Analytics E170 NT-proBNP immunoassay (Roche Diagnostics, Mannheim, Germany). During the hospital stay, a routine biochemical workup was performed, including creatinine, hemoglobin, LDL cholesterol, glycated hemoglobin (HbA1C), sodium, potassium, and peak troponin T. The four-variable Modification of Diet in Renal Disease (MDRD) procedure was used to calculate the estimated rate of the glomerular filtration (eGFR) [12].

### 2.5. PCI

The PCI was performed, in agreement with the established procedures, as soon as possible after the confirmation of the AMI, without surpassing the 12 h interval for STEMI and high-risk NSTEMI patients. A stenosis of more than 75% in the right coronary, circumferential, or anterior descending artery, or and more than 50% in the left main coronary trunk, was considered notable. The presence of notable stenosis in more than one coronary vessel was characterized as multivessel coronary artery disease (CAD). A TIMI flow of grade 3 at the infarct-related artery deemed the PCI as successful.

### 2.6. Echocardiography

Initial echocardiography was performed in the hospital, at 1.3 ± 0.6 days following the PCI, using a GE Vivid E7 ultrasound system (GE Health Medical, Milwaukee, WI, USA). The follow-up echocardiographic examination was conducted at 24 months after the initial exam.

#### 2.6.1. Conventional Echocardiography

LV diameters, volumes, and LVEF were determined using Simpson’s biplane method, as recommended by the American Society of Echocardiography [13]. A 17-segment model was used to visually assess regional wall motion, with each segment recorded as 5—aneurysm, 4—dyskinesia, 3—akinesia, 2—hypokinesia, and 1—normal. The WMSI was obtained by averaging the segmental results. Doppler exam of the trans mitral valve in diastole was used to assess the diastolic performance of the LV, and the E/A ratio was calculated (E—maximal proto diastolic velocity; A—maximal tele diastolic velocity) [14]. Any increase in LVEDV >20% from baseline was considered as LV remodeling [2].

#### 2.6.2. Two Dimensional-Speckle Tracking Echocardiography (2D-STE)

**Figure 1:**
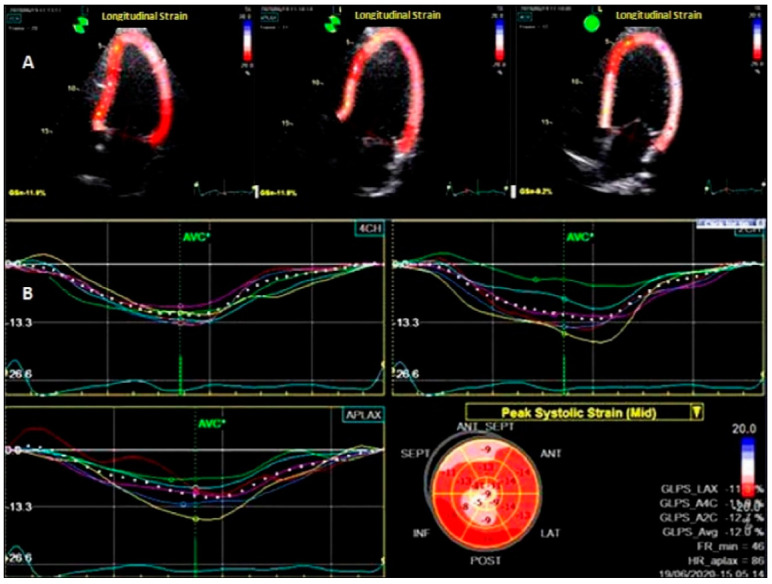
Two–dimensional speckle–tracking echocardiography at the level of the left ventricle. (**A**) Longitudinal analysis of deformation in apical 2-, 3-, and 4 chamber views; (**B**) bull’s eye map.

To assess LV deformation patterns, 2D-STE was used [8,15]. Offline analysis was performed with the Echo PAC system, version 204 (GE Ving med). Global strain and strain rate were measured in three directions: longitudinal (L), radial (R), and circumferential (C). From the short-axis images at the basal, middle levels, and apical incidences, the LV radial and circumferential strains and strain rates (LVRS, LVRSR, LVCS, LVCSR,) were assessed. From the 2-, 4-, and 3-chamber apical views, the left ventricular longitudinal strain (LVLS) and strain rates (LVSR) were calculated. The software detected the myocardium automatically; however the cardiac border was manually adjusted to increase the performance. Moreover, every apical and short-axis view was split into six parts, for a total of 18 segments. The systolic strain rates were calculated in three sections: radial (RSR), circumferential (CSR), and longitudinal (LSR). The average of the 18 segments yielded the global peak circumferential strain (GCS), the global radial strain (GRS), and the global peak longitudinal strain (GLS), as shown in Figure 1

### 2.7. Statistical Analysis

Numbers and percentages were used to represent categorical variables. Continuous variables with normal distributions were reported as means ± 1 standard deviation, while those with non-normal distribution were reported as the median (25th, 75th percentile), as per Kolmogorov–Smirnov tests.

The paired t-test was used to compare differences between the two patient groups for continuous and normally distributed parameters: the Mann–Whitney U-test was used for continuous non-normally distributed parameters, and the chi-squared test was used for categorical parameters. Univariate analysis was used to determine the odds ratio (OR) and confidence interval (CI) of several characteristics linked to LV remodeling. Univariately significant variables related to LV remodeling were included in the multivariate analysis using a forward stepwise logistic regression model. The receiver operating characteristic (ROC) curve was used to assess the sensitivity and specificity of the independent predictors found via multivariate logistic regression. The Kaplan–Meier method was used for survival analyses, and log-rank tests were used to examine differences among groups. For statistical analysis, we used the MedCalc Statistical Software, version 19.6. (MedCalc Software, Ostend, Belgium). *p* < 0.05 two-tailed values were considered statistically significant.

## 3. Results

A total of 9 of the 253 AMI who were initially included were removed and not analyzed because they underwent a second PCI during the 2-year follow-up period. Finally, the study group included 244 patients ranging in age from 32 to 74 years (mean age 66.4 ± 13.3 years), with 185 (73%) men.

At the end of the 2-years follow-up period, we found that 58 (26%) of the patients had an LVEF <50% (Table 1). The patients in whom LVEF decreased were older, more often hypertensive and with a history of smoking, presented more often with a Killip class >2, higher peak creatine kinase MB levels, and more often exhibited multi-vessel coronary artery disease. The discharge medication did not differ significantly between the groups.

Throughout the 2-year follow-up period, 19 (8%) of the patients were hospitalized for HF; no patient died. The hospitalization rate was 6% in group I, and 12% in group II (*p* < 0.0001).

The echocardiographic findings are presented in Table 2. At baseline, Group II patients had smaller LV end-diastolic and end-systolic volumes, as well as lower infarct-related longitudinal strains and strain-rates. At the 24-month evaluation, Group II had higher LV end-systolic and end-diastolic volumes, wall motion score indexes, as well as smaller global longitudinal strains and strain rates. The rate of LV remodeling was significantly higher in group II (86% vs. 28%, *p* < 0.0001).

During the 2-year follow-up, no patient died. A total of 19 (8%) were hospitalized due to heart failure, 12 (6%) from Group I and 7 (12%) from Group II (*p* < 0.0001).

**Figure 2:**
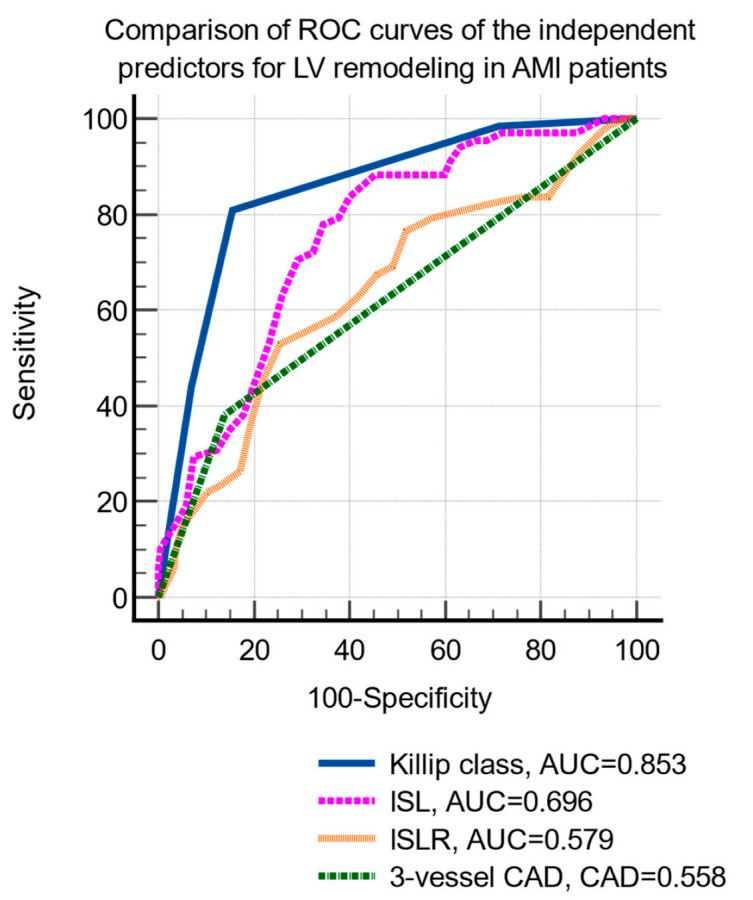
Comparison of receiver operating characteristic curves of the independent parameters predictive of LV remodeling. Abbreviations: LVEF, left ventricular ejection fraction; AMI, acute myocardial infarction; ROC, receiver operating characteristic; CAD, coronary artery disease; ILS, infarct-related longitudinal strain; ILSR, infarct-related longitudinal strain rate.

LV remodeling was significantly associated with age, hypertension, hypercholesterolemia, smoking status, Killip class, peak creatin-kinase MB level, two- and three-vessel coronary artery disease, and infarct-related longitudinal strain and strain rate (Table 3). The multivariate logistic regression identified the following independent predictors of LV remodeling: Killip class, three-vessel coronary artery disease, infarct-related strain, and strain rate (Table 4). The comparison of the ROC curves of the independent prediction of LV remodeling is shown in Figure 2.

The cutoff values that were identified by ROC curve analysis were: Killip class >2 (sensitivity 80.9%, specificity 84.3%, *p* < 0.001), ILS <−12.8% (sensitivity 83.8%, specificity 60%, *p* < 0.001) and ILSR ≤0.7/s (sensitivity 52.9%, specificity 74.6%, *p* < 0.001), and the presence of 3-vessels CAD (sensitivity 38.2%, specificity 85.9%, *p* = 0.004).

HF hospitalizations were significantly associated using univariate analysis, with Killip class, the baseline values of NT-proBNP, the infarct related longitudinal strain and the presence of LV remodeling at 24-months (Table 4). Using multivariate analysis, Killip class, three-vessel infarct-related longitudinal strain, and LV remodeling were identified as independent predictors for the risk of HF hospitalizations (Table 4).

**Figure 3:**
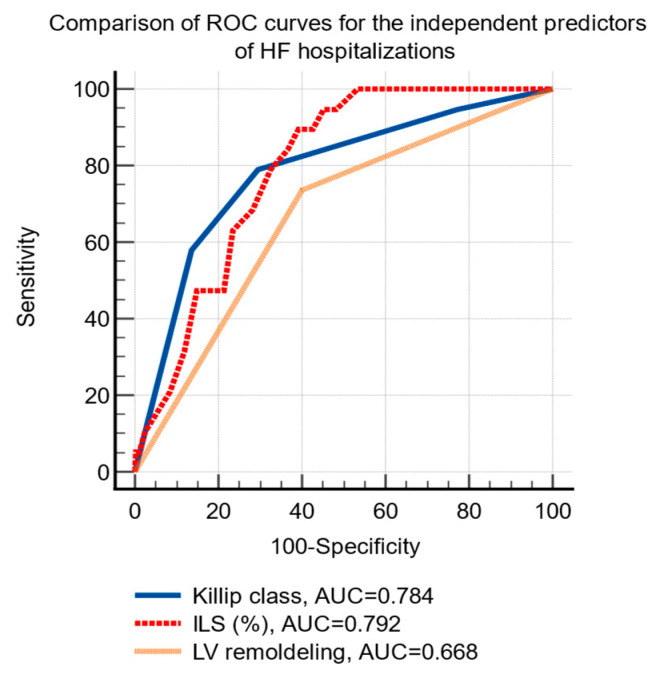
Comparison of receiver operating characteristic curves of the independent predictors of hospital admissions due to HF after an acute myocardial infarction. Abbreviations: HF, heart failure; ROC, receiver operating characteristic; AUC, area under the curve; ILS, infarct-related longitudinal strain; LV, left ventricle.

The ROC curve analysis established the following cutoff levels for the independent predictors of HF hospitalizations: Killip class >2 (sensitivity 78.9%, specificity 70.5%, *p* < 0.001) and infarct-related longitudinal strain ≤−12.5% (sensitivity 90%, specificity 61%, *p* < 0.001), the presence of LV remodeling (sensitivity 73.7%, specificity 60%, *p* < 0.01), Figure 3.

**Figure 4:**
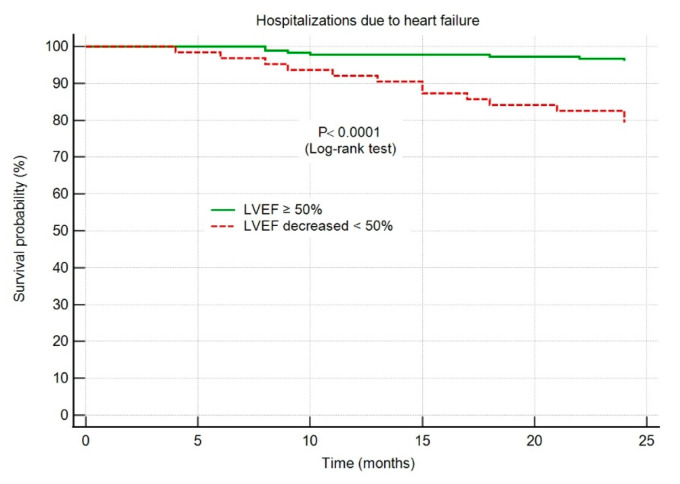
Kaplan–Meier survival curves showing hospitalizations due to heart failure. Abbreviations: HF, heart failure; AMI, acute myocardial infarction; LVEF, left ventricular ejection fraction.

The HF hospitalization-free survival probability was significantly higher in group I patients, with an LVEF maintained ≥50% throughout the 2-year follow-up period, as shown in Figure 4.

## 4. Discussion

Our case-control observational analysis is the first study in Romania to examine the predictive usefulness of 2D-STE for the early detection of LV remodeling in AMI patients with a normal LVEF following a successful reperfusion by PCI. The study was carried out by two teams of researchers from western Romania and showed that post-MI heart failure is still a problem in the contemporary era of myocardial reperfusion therapies, demanding modern therapeutic strategies.

In comparison to pharmaceutical thrombolysis, PCI patients appear to have an improvement in left ventricular (LV) function, despite the presence of LV remodeling [2,16]. Because of this, it is important to find patients who are at risk of LV remodeling among those with a preserved LVEF after an AMI that was successfully treated with PCI.

Our study showed that, even in AMI patients with LVEF ≥50% after a successful PCI performed within the first 12 h from the onset of symptom, adverse LV remodeling and HF still emerge. At the 24-month evaluation, we found that the majority (74%) of AMI patients experienced an improvement in LV systolic function, 26% suffered a decline in LVEF below 50%, and 8% suffered HF hospitalizations. The discharge medication was similar in both groups. No survival differences were observed among the two patient groups. Adverse LV remodeling was found in 41% of patients, and was seen significantly more frequently among those with a decrease in LVEF (86% vs. 21%, *p* < 0.0001).

The frequency of LV post-MI remodeling depends on the definition used, as well as on the imaging modality that was applied. When left ventricular post-MI remodeling is defined as a sonographic increase in LVEDV of more than 20%, and when different temporal patterns are recognized, frequencies of 42% to 48% are reported [2,17]. When LV- post MI remodeling is defined merely at a given time point, it is significantly less prevalent (less than 40%), [18]. Most studies assess the incidence of increased LVEDV at one year after the AMI.

However, several studies suggest that post-MI LV remodeling is an ongoing process that continues at least up to 2 years after the AMI [19,20]. Further, apart from adverse LV remodeling, reverse LV remodeling also occurs, explaining the improvement in LV function. Reverse LV remodeling is defined by a decrease in LV end-systolic volume (ESV) >10% [21].

In patients who have had an AMI, either a STEMI or an NSTEMI, the LVEF is an important factor in determining the outcome [22]. Since it can detect a variety of subtle disorders that are linked to a bad outcome, echocardiography represents an important approach to evaluate the LVEF. In our study, the risk of HF hospitalization during the 2-year follow-up in post-MI patients was 5.8 times higher among the patients in whom LVEF decreased below 50% (95% CI 2.3–14.8, *p* < 0.001) and 3.8 times higher among those with echocardiographic signs of adverse LV remodeling (95% 1.4–10.9, *p* < 0.01).

Previous research has shown that speckle-tracking imaging has the capability of evaluating the regional function of the LV [23]. It has been proven that a decreased global longitudinal strain improves the prognostic evaluation in HF patients with a reduced systolic function [24]. An accurate and non-invasive predictor of left ventricular functional deterioration in AMI patients is the global longitudinal strain measured. This was shown to be more precise than LVEF and WMIS, particularly in AMI patients with a LVEF higher than 40% [22]. Ersboll’s research showed that a decreased GLS was strongly associated with a poor outcome in AMI patients who had preserved LVEF. It is possible that timely assessment of GLS in this cohort could be used as a risk classification instrument for additional supervision in clinical research [25]. The GLS was independently linked to the development of in-hospital established HF (*p* = 0.003) in a clinical trial that involved 153 AMI patients with LVEF over 55% at 2.5 days following a successful PCI. The authors hypothesized that by incorporating GLS into a screening model, it would be possible to improve the accuracy of predicting clinical heart failure following AMI [26]. Our study revealed that the decrease in LVEF <50% in post-MI patients was significantly predicted by a reduced strain (<−12.8%) and strain rate (<0.7/s) of the infarct-related segments, at baseline. The post-MI patients with a decrease in LVEF below 50% did not differ in survival from those with LVEF ≥50%, but had significantly more HF admissions during the 2-year follow-up period.

Van de Bijl et al. [17] also investigated LV post-infarct remodeling by echocardiography, including 1.995 STEMI patients who were followed-up for 8 years. Using the increase in LVEDV >20% from baseline as a criterion for remodeling, they found that 48% of them were remodelers in the first 12 months after the primary PCI. Remodelers did not differ from non-remodelers in survival, but had a higher rate of HF admissions.

Hassell et al. [21] evaluated LV post-MI remodeling in 155 STEMI patients revascularized by primary PCI using cardiac magnetic resonance at baseline, at 4, and at 24 months. The 24-month CMR showed that 50% of the patients had improved LVEF, and 50% had a decreased LVEF compared to the one determined at 4 months. The authors concluded that both positive and negative LV post-MI remodeling reflect an ongoing process that lasts up for a minimum of 2 years. Deterioration of LVEF was associated with higher LV end-systolic volumes and less wall-thickening in the remote segments. Improvement of LVEF was associated with smaller LV end-systolic volumes and greater wall thickening in the infarct and non-infarct areas. There were no differences among the two groups regarding mortality and HF admissions during a follow-up period of 5 years.

In another study that investigated LV post-infarct remodeling with CMR after primary PCI, after a mean follow-up of 6 years, the primary endpoint (cardiovascular mortality, heart failure hospitalization, or ventricular arrhythmias) was achieved in 13% of patients [27].

The higher rate of HF hospitalization in LV post-infarct remodelers suggests the need to intensify preventive strategies in this group, for example, by using speckle-tracking imaging (STI) to detect early subtle changes in LV function. Another preventive strategy could be the use of an angiotensin receptor neprilysin inhibitor (ARNI) [28], as it could prevent and lessen the decline of LVEF in post-MI patients [29,30].

EMPACT-MI and DAPA-MI are two large trials now evaluating sodium–glucose cotransporter 2 (SGLT2) inhibition in patients at risk for HF after MI. They are also investigating whether empagliflozin or dapagliflozin versus placebo improves the time to first HF hospitalization or CV death after an AMI [31,32,33].

In the acute MI phase, treatment with the anti-ischemic drug trimetazidine may restore energy supply, minimize mitochondrial damage, and limit ischemia/reperfusion harm. This medication is currently being studied to see if it can protect against persistent post-MI remodeling [34].

This paper brings valuable data to the limited information regarding LV remodeling after successful myocardial revascularization by PCI, using a modern imagistic method to detect subclinical LV systolic dysfunction. We believe that our observational prospective study can build the groundwork for a randomized trial to evaluate latent LV dysfunction after AMI, as well as the efficacy of new drugs that could prevent and mitigate post-MI heart failure. According to the findings of this investigation, unfavorable LV remodeling following an acute myocardial infarction should be monitored for at least 2 years.

Study limitations: the study has a number of limitations. This was a prospective, two-center research. We included only AMI patients at sinus rhythm and having a LVEF ≥50% following PCI, likely presenting small extent infarctions. Although it is known that myocardial contractility may improve within 2 days following revascularization, the earliest possible time after the PCI was 1.3 ± 0.6 days. These findings, on the other hand, lend support to the utilization of speckle-tracking imaging in the process of risk classification for AMI patients.

## 5. Conclusions

Heart failure still occurs in AMI patients with normal LVEF after a successful PCI for AMI. We found that 26% of these patients experienced a decrease in LVEF below 50% during the 2-year follow-up, 41% with evidence of LV remodeling, and about 8% required hospitalization for HF. No differences in survival were noted.

The study demonstrates the effectiveness of 2D-speckle tracking imaging, a more sensitive technique that allows for more efficient risk classification and preventative therapy in AMI patients to minimize pathological LV remodeling and HF.

## Figures and Tables

**Table 1:** Characteristics of AMI patients split for LVEF at 24 months.

AMI Patients with LVEF ≥50% after PCI	Group I (LVEF ≥50%) (*n* = 186)	Group II LVEF Decrease (<50%) (*n* = 58)	Overall (*n* = 244)	*p* Value
At baseline				
STEMI/NSTEMI (*n*, %)	165 (89)/21 (11)	55 (94)/5 (6)	220 (96)/24 (17)	0.27
Age (years)	64.8 ± 12	72.0 ± 13	66 ± 13	**<0.0001**
Male sex (*n*, %)	130 (70)	48 (82)	178 (73)	0.06
Diabetes mellitus	104 (56)	34 (58)	138 (56)	0.78
Systemic hypertension	117 (63)	54 (94)	171 (70)	**<0.0001**
Hypercholesterolemia	138 (74)	50 (86)	188 (77)	0.05
Smoking history	65 (35)	30 (50)	95 (39)	**0.04**
Obesity	52 (28)	12 (20)	64 (28)	0.22
SBP (mmHg)	128.0 ± 12.1	145.6 ± 13.8	129.8 ± 27.0	**<0.0001**
DBP (mmHg)	79.3 ± 6.5	86.7 ± 13.2	75.9 ± 15.9	**<0.0001**
Heart rate (beats/min)	82 ± 17	79 ± 21	80 ± 19	0.20
Chronic renal failure	50 (27)	24 (29)	74 (30)	0.76
Killip class				
I (%)	52 (28)	1 (2)	53 (22)	**<0.0001**
II (%)	106 (57)	10 (18)	116 (47)	**<0.0001**
III (%)	17 (9)	28 (49)	45 (20)	**<0.0001**
IV (%)	11 (6)	18 (31)	29 (12)	**<0.0001**
Peak CPK-MB (IU/L), median (25th, 75th percentile)	172 (67.2, 332.5)	258 (81, 498.25)	187 (77, 393)	**<0.01**
NT-proBNP (ng/L) median (25th, 75th percentile)	220 (60, 307)	900 (210, 2450)	220 (80, 500)	0.64
eGFR (mL/min/1.73 m^2^)	75.8 ± 21.7	65.8 ± 23.0		**<0.01**
Culprit vessel				
LAD (%)	95 (51)	22 (38)	97 (40)	0.07
LCX (%)	22 (12)	8 (14)	30 (12)	0.68
RCA (%)	69 (37)	24 (42%)	93 (41)	0.12
Coronary artery disease				
1-vessel (%)	126 (68)	13 (22)	139 (62)	**<0.0001**
2-vessel (%)	32 (17)	19 (33)	52 (23)	**<0.01**
3-vessel (%)	16 (15)	26 (45)	42 (19)	**<0.0001**
Medication at discharge				
ACEI or ARB	151 (81)	45 (78)	196 (80)	0.60
Beta-blocker	147 (79)	44 (76)	191 (78)	0.62
Calcium antagonists	41 (22)	14 (24)	55 (23)	0.74
Statins	138 (74)	42 (72)	180 (74)	0.75
At 24-months follow-up				
Hospitalizations for heart failure	12 (6)	7 (12)	19 (8)	**<0.0001**
Repeated PC	6 (3)	3 (5)	9 (4)	0.70
CABG	0 (0)	0 (0)	0 (0)	1.00
Deaths	0 (0)	0 (0)	0 (0)	1.00

**Notes:** Continuous variables that are normally distributed are presented as mean ± 1 standard deviation; continuous variables that are not normally distributed are presented as median (25th, 75th percentile). Statistically significant values are shown in bold (*p* < 0.05). Abbreviations: DBP, diastolic blood pressure; SBP, systolic blood pressure; eGFR, estimated glomerular filtration rate; ARB, angiotensin receptor blocker; CPK-MB, creatine kinase MB isoenzymes; BNP, brain natriuretic peptide; IU, international units; LAD, left anterior descending artery; RCA, right coronary artery; LCX, left circumflex artery; ACEI, angiotensin converting enzyme inhibitor.

**Table 2:** Echocardiographic findings.

AMI Patients with LVEF ≥ 50% after PCI	LVEF ≥ 50% *n* = 186	LVEF Decrease (<50%) *n* = 58	Overall *n* = 244	*p* Value
**Baseline**				
LVEF (%)	58.5 ± 8.3	56.3 ± 5.5	57.2 ± 7.1	0.16
LVEDV (mL)	105 ± 17	95 ± 18	100 ± 17	**0.0001**
LVESV (mL)	43 ± 8.5	38 ± 9.6	40.5 ± 9.1	**0.0001**
Stroke volume index (mL/m^2^)	41 ± 10.4	39.6 ± 11.4	40.3 ± 10.8	0.37
E/A ratio	1.10 ± 0.42	1.06 ± 0.48	1.08 ± 0.46	0.53
WMSI	2.25 ± 0.21	2.29 ± 0.26	2.27 ± 0.24	0.22
GLS (%)	−18.8 ± 3.8	−17.9 ± 3.3	−18.3 ± 3.6	0.09
GLSR (s^−1^)	−1.12 ± 0.19	−1.07 ± 0.21	−1.10 ± -.20	0.08
GCS (%)	−17.4 ± 4.0	−17.2 ± 4.8	−17.3 ± 4.2	0.74
GCSR (s^−1^)	−1.44 ± 0.39	−1.46 ± 0.38	−1.45 ± 0.39	0.72
GRS (%)	36.5 ± 12.2	37.2 ± 12.3	36.9 ± 12.2	0.69
GRSR (s^−1^)	1.82 ± 0.41	1.73 ± 0.37	1.78 ± 0.40	0.12
ILS(%)	−13.1 (12.4, 14.00)	−12.3 (11.6, 12.8)	−12.9 (12.2, 13.8)	**<0.0001**
ILSR (s^−1^)	−0.76 (0.70, 0.80)	−0.74 (0.69, 0.80)	−0.74 (0.69, 0.80)	**0.0004**
Number of infarct-related segments	5.6 ± 4.7	6.3 ± 5.2	5.9 ± 4.9	0.32
**After 24 months**				
LVEF (%)	62.5 ± 7.1	49.5 ± 8.0	61.1 ± 7.6	**<0.01**
LVEDV (mL)	104 ± 26	112 ± 32	109.5 ± 28	0.19
LV Remodeling (*n*,%)	52 (28%)	50 (86%)	102 (41)	**<0.0001**
LVESV (mL)	41 ± 15	51 ± 12	44 ± 13	**<0.01**
Stroke volume index (mL/m^2^)	48.2 ± 5.6	44.5 ±7.4	46.85 ± 6.5	**<0.01**
E/A ratio	1.10 ± 0.32	1.03 ± 0.28	1.09 ± 0.30	0.08
WMSI	1.97 ± 0.5	2.18 ± 0.4	2.07 ± 0.5	**<0.01**
GLS (%)	−21.2 ± 3.5	−18.4 ± 4.1	−18.8 ± 3.7	**<0.0001**
GLSR (s^−1^)	−1.62 ± 0.21	−1.02 ± 0.24	−1.08 ± 0.23	**<0.0001**
GCS (%)	−19.6 ± 4.7	−18.5 ± 4.2	−19.1 ± 4.4	0.1
GCSR (s^−1^)	−1.41 ± 0.30	−1.36 ± 0.28	−1.38 ± 0.29	0.2
GRS (%)	33.7 ± 14.8	31.7 ± 14.5	34.6 ± 0.43	0.37
GRSR (s^−1^)	1.60 ± 0.42	1.49 ± 0.45	1.60 ± 0.43	0.08

**Notes:** Values are presented as mean ± 1 standard deviation. Statistically significant values are shown in bold (*p* < 0.05). Abbreviations: LVEDV, left ventricular end-diastolic volume; LVESV, left ventricular end-systolic volume; LVEF, left ventricular ejection fraction; WMSI, wall motion score index; E, early diastolic wave velocity; A, late diastolic wave velocity; GCSR, global circumferential strain rate; GCS, global circumferential strain; GLSR, global longitudinal strain rate; GRS, global radial strain; GLS, global longitudinal strain; GRSR, global radial strain rate; LSR, longitudinal strain rate; LS, longitudinal strain; ILS, infarct-related longitudinal strain; ILSR, infarct-related longitudinal strain rate.

**Table 3:** Predictors for LV remodeling in AMI patients with LVEF ≥ 50% after successful PCI.

Univariate Logistic Regression	Odds Ratio	95% CI	*p* Value
Age (years)	1.03	1.01–1.06	**0.0037**
Systemic hypertension	3.67	1.38–9.73	**0.0029**
Hypercholesterolemia	3.9	1.70–9.16	**0.0014**
Smoking	0.4	0.24–0.80	**0.0077**
Killip class	3.94	1.70–9.16	**0.0003**
Peak CPK-MB (IU/L)	1.03	1.07–1.02	**<0.0001**
2- vessel CAD	2.1	1.12–4.21	**0.0187**
3-vessel CAD	3.6	1.90–6.97	**<0.0001**
ILS (%)	5.27	3.43–8.08	**<0.0001**
ILSR (s^−1^)	0.04	0.01–0.13	**<0.0001**
**Multivariate Logistic Regression**	**Odds Ratio**	**95% CI**	***p* Value**
Killip class	17.58	3.97–77.76	**0.0002**
3-vessel CAD	2.73	1.06–7.05	**0.0375**
ILS (%)	5.77	2.90–11.46	**<0.0001**
ILSR (s^−1^)	0.11	0.03–0.39	**0.0005**

**Notes:** Values are presented as mean ± 1 standard deviation. Statistically significant values are highlighted in bold (*p* < 0.05). Abbreviations: CPK-MB, creatine phosphokinase kinase MB isoenzymes; DBP, diastolic blood pressure; SBP, systolic blood pressure; IU, international units; CAD, coronary artery disease; HLSR, harmed longitudinal strain rate; HLS, harmed longitudinal strain; LVEDV, left ventricular end-diastolic volume.

**Table 4:** Predictors for heart failure hospitalizations during the 2-year follow-up after an AMI.

	Univariate Logistic Regression			Multivariate Logistic Regression		
	Odds Ratio	95% CI	*p* Value	Odds Ratio	95% CI	*p* Value
Killip class	2.91	1.74–4.85	**<0.0001**	2.65	1.54–4.56	**<** **0.001**
NT-pro BNP (ng/L)	1.00	1.00–1.00	**0.01**			
ILS (%)	0.24	0.11–0.51	**<** **0.001**	0.26	0.11–0.57	**<** **0.001**
LV remodeling	4.2	1.46–12.04	**0.004**	4.5	1.36–14.86	**0.01**

**Notes:** Statistically significant values are highlighted in bold (*p* < 0.05). Abbreviations: CAD, coronary artery disease; BNP, brain natriuretic peptide; ILS, infarct-related longitudinal strain; LV, left ventricle.

## Data Availability

Not applicable.
